# Research hotspots and trends on neuropathic pain-related mood disorders: a bibliometric analysis from 2003 to 2023

**DOI:** 10.3389/fpain.2023.1233444

**Published:** 2023-12-21

**Authors:** Xiaohua Wang, Yueyang Zhuang, Zhigang Lin, Shuijin Chen, Lechun Chen, Hongye Huang, Hui Lin, Shiye Wu

**Affiliations:** ^1^ College of Rehabilitation Medicine, Fujian University of Traditional Chinese Medicine, Fuzhou, Fujian, China; ^2^ Affiliated Rehabilitation Hospital of Fujian University of Traditional Chinese Medicine, Fuzhou, Fujian, China; ^3^ Fujian Key Laboratory of Rehabilitation Technology, Fuzhou, Fujian, China

**Keywords:** neuropathic pain, mood disorders, CiteSpace, bibliometric analysis, Web of Science

## Abstract

**Introduction:**

Neuropathic Pain (NP) is often accompanied by mood disorders, which seriously affect the quality of life of patients. This study aimed to analyze the hotspots and trends in NP-related mood disorder research using bibliometric methods and to provide valuable predictions for future research in this field.

**Methods:**

Articles and review articles on NP-related mood disorders published from January 2003 to May 2023 were retrieved from the Web of Science Core Collection. We used CiteSpace to analyze publications, countries, institutions, authors, cited authors, journals, cited journals, references, cited references, and keywords. We also analyzed collaborative network maps and co-occurrence network maps.

**Results:**

A total of 4,540 studies were collected for analysis. The number of publications concerning NP-related mood disorders every year shows an upward trend. The United States was a major contributor in this field. The University of Toronto was the most productive core institution. C GHELARDINI was the most prolific author, and RH DWORKIN was the most frequently cited author. PAIN was identified as the journal with the highest productivity and citation rate. The current research hotspots mainly included quality of life, efficacy, double-blind methodology, gabapentin, pregabalin, postherpetic neuralgia, and central sensitization. The frontiers in research mainly focused on the mechanisms associated with microglia activation, oxidative stress, neuroinflammation, and NP-related mood disorders.

**Discussion:**

In conclusion, the present study provided insight into the current state and trends in NP-related mood disorder research over the past 20 years. Consequently, researchers will be able to identify new perspectives on potential collaborators and cooperative institutions, hot topics, and research frontiers in this field.

## 1. Introduction

Pain is an unpleasant feeling and emotional experience associated with actual or potential tissue damage (1). This definition emphasizes that pain is a comprehensive phenomenon involving sensory, emotional, and cognitive factors. Neuropathic pain (NP) is a type of long-term chronic pain caused by damage to the somatosensory nervous system resulting from a variety of diseases, and NP is a complex pathophysiological process (2, 3). There are many possible causes of NP, such as injury, surgery, diabetes, infection, and cancer (4). NP usually manifests as spontaneous pain, hyperalgesia, and paresthesia (5). Studies have shown that the occurrence, development, persistence, and aggravation of chronic NP are closely related to psychological factors such as anxiety, depression, fear, and stress (6). Research has found that patients with NP often experience accompanying emotional issues such as depression, anxiety, anger, and social isolation. These emotional disorders are closely related to the severity and duration of the pain, forming a complex interplay between them (7, 8). NP not only affects patient sleep and work but also increases the incidence of depression, anxiety, and other affective disorders. It seriously affects the quality of life and brings a great burden to patients and society (9–11). In order to solve this problem, studies have focused on the mechanisms connecting pain sensitivity and mood disorders (12–14). Patients with NP have unpleasant emotional and experiences related to pain over a long time. The harm caused by these emotions and psychological problems is often more serious than the pain itself, and the negative emotions caused by pain often further aggravate the pain experience of patients, increase the degree of pain, and worsen prognosis (15, 16).

Bibliometrics is a branch of informatics that conducts quantitative analysis of scientific literature in order to understand the emerging trends and knowledge structures in a field of research (17). Bibliometrics can use keyword co-occurrence networks and keyword emergences to track topic hotspots and research frontiers (18). CiteSpace is an information visualization software, which can detect the classic literature, research hotspots and frontiers of the discipline by measuring the literature in a specific field (19). CiteSpace is related to three core concepts: emergent word detection, mediation centrality, and co-occurrence networks. These concepts can solve three practical problems, namely the identification of research status, hotspots, and frontiers. Because the software uses visualization to show the structure, rules, and distribution of scientific knowledge, the visualization results obtained through CiteSpace analysis are also called “scientific knowledge maps”. The application of bibliometrics in medicine is an essential research method that allows a deeper understanding of the current status and trends in the medical field, assessment of academic impact, identification of knowledge evolution and innovative paths, promotion of interdisciplinary collaboration, provision of scientific support for medical policies and practices, optimization of knowledge management and resource integration, and continuous advancement of medical research and healthcare practices (20). CiteSpace can be used to discover and visualize emerging academic achievements, key paths, and research trends in the research of NP-related mood disorders, and this utility plays an important role in exploring the connotations and developments of related research fields. Despite the significant impact of NP on mood disorders and the growing interest in the field, there is a notable lack of studies utilizing visualization techniques to explore and understand the intricate relationship between NP and mood disorders. In the context of NP-related mood disorders, visualization can potentially offer a unique perspective in comprehending the interplay between physiological, psychological, and social factors. However, visualization research on NP-related mood disorders is very limited. Therefore, this paper analyzed the literature on NP-related mood disorders using CiteSpace software, generating a corresponding visual atlas and identifying and displaying the new trends and dynamics of scientific development; it additionally provides a data reference for future research.

## 2. Materials and methods

The search strategy is built as follow: TI = (“neuropathic pain” OR “neuralgia”) AND TI = (“emotions” OR “emotional distress” OR “emotional disorder” OR “mood disorders” OR “anxiety” OR “depression” OR “stress” OR “emotional regulation” OR “emotional processing” OR “emotional control” OR “emotion regulation” OR “negative affect” OR “psychological factors” OR “psychosocial factors”) in the Web of Science Core Collection. The search covered the relevant literature published between January 1, 2003, and May 1, 2023. The literature type was chosen to articles and review articles, and the language was set as English. A total of 4,869 publications were retrieved, and irrelevant and repetitive publications were deleted to ensure the accuracy and validity of the data. Finally, a total of 4,540 publications were included.

Microsoft Excel software (v2016; Microsoft Corporation, Redmond, WA, USA) was used to conduct a descriptive statistical analysis of the annual publication numbers and draw a linear curve. CiteSpace (5.7R5) was used to import the selected literature in the format of “full records and cited references”. Time-slicing was performed from January 2003 to May 2023 (1 year per slice), and we set the node type (country, institution, author/cited author, journal/cited journal, references/cited reference, keyword), selection criteria (g-index: *k* = 5), and pruning (pathfinder, pruning sliced network). The software values for other components were set to their default settings.

CiteSpace generates co-occurrence network graphs wherein each node is depicted as a circle. The size of the circle corresponds to the frequency of the node, with larger circles indicating higher frequency. The size of the nodes can reflect the frequency of the keywords or themes in the literature, with larger nodes indicating a higher number of occurrences in the literature and representing relatively more important keywords or themes. The color of the circle represents the time of node appearance, with darker hues indicating earlier appearance. The connections established among nodes serve as indicators of collaboration, co-occurrence, or co-referencing relationships. Tighter connections may indicate stronger associations between nodes, and these nodes in the research field may have similar research directions or themes. The thickness of the purple ring denotes the level of centrality, and nodes exhibiting high centrality values (>0.1) are typically regarded as pivotal or transformative elements within a given field (17). Co-occurrence analysis, cluster analysis, and co-citation analysis were performed on different node types.

## 3. Results

### 3.1. Analysis of annual publications

**Figure 1:**
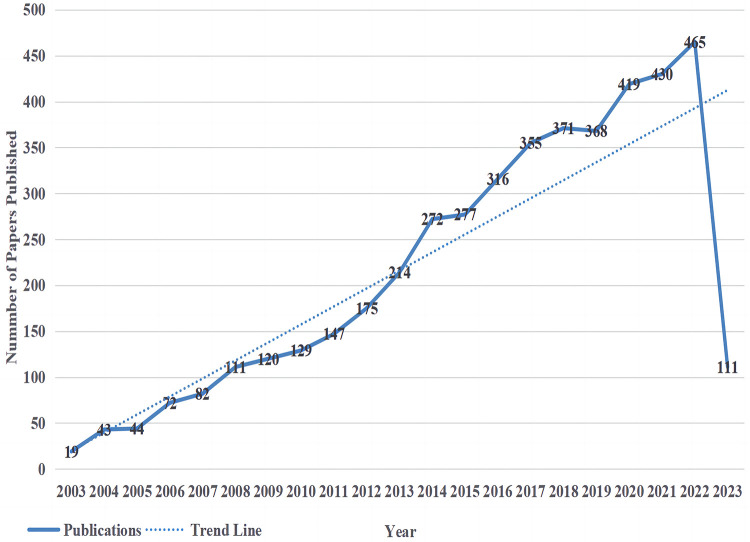
The annual number of publications related to NP-related mood disorders.

The trends in publishing are an important indicator to measure the development of research in a certain field. Therefore, drawing distribution curves of the number of publications over time can effectively evaluate the research status of a discipline and further predict its development trends. Figure 1 shows the annual distribution of research-related literature in the field of NP associated with mood disorders in the Web of Science Core Collection. A total of 4,540 publications were retrieved, with an average annual publication volume of 227. On the whole, the number of publications has increased but fluctuated. It could be roughly divided into three research stages. (1) The initial stage (2003–2007), where research on NP associated with mood disorders had just started, belonged to the embryonic stage of this field, with an average annual number of 52 articles published; the academic community did not pay much attention to this field during this stage. (2) In the stable growth period (2008–2013), the research on NP-related mood disorders entered a stable period of development, and the average number of published papers reached 149, which showed that the research in this field gradually received more attention, and its influence on the current academic and practical circles gradually increased. (3) The rapid growth period (2014–2023), with an average annual number of 338 papers, was the outbreak period of NP-related mood disorder research, indicating that research in this field was attached great importance to researchers at this stage. During this period, the research on NP-related mood disorders made unprecedented progress, developing a certain system and structure. Scholars have begun to focus on stable research directions and research has maintained a stable growth rate. Although the number of papers published in 2023 was only calculated until May, it is expected that 2023 and even the next few years will also show an upward trend. The past 20 years was divided into four periods (Supplementary Figure 1). The most cited period per paper (248 times) was from 2018 to 2023; the number of articles published in this period reached 2,340, and the total number of citations (95,600 times) was at its highest.

### 3.2. Analysis of countries

**Figure 2:**
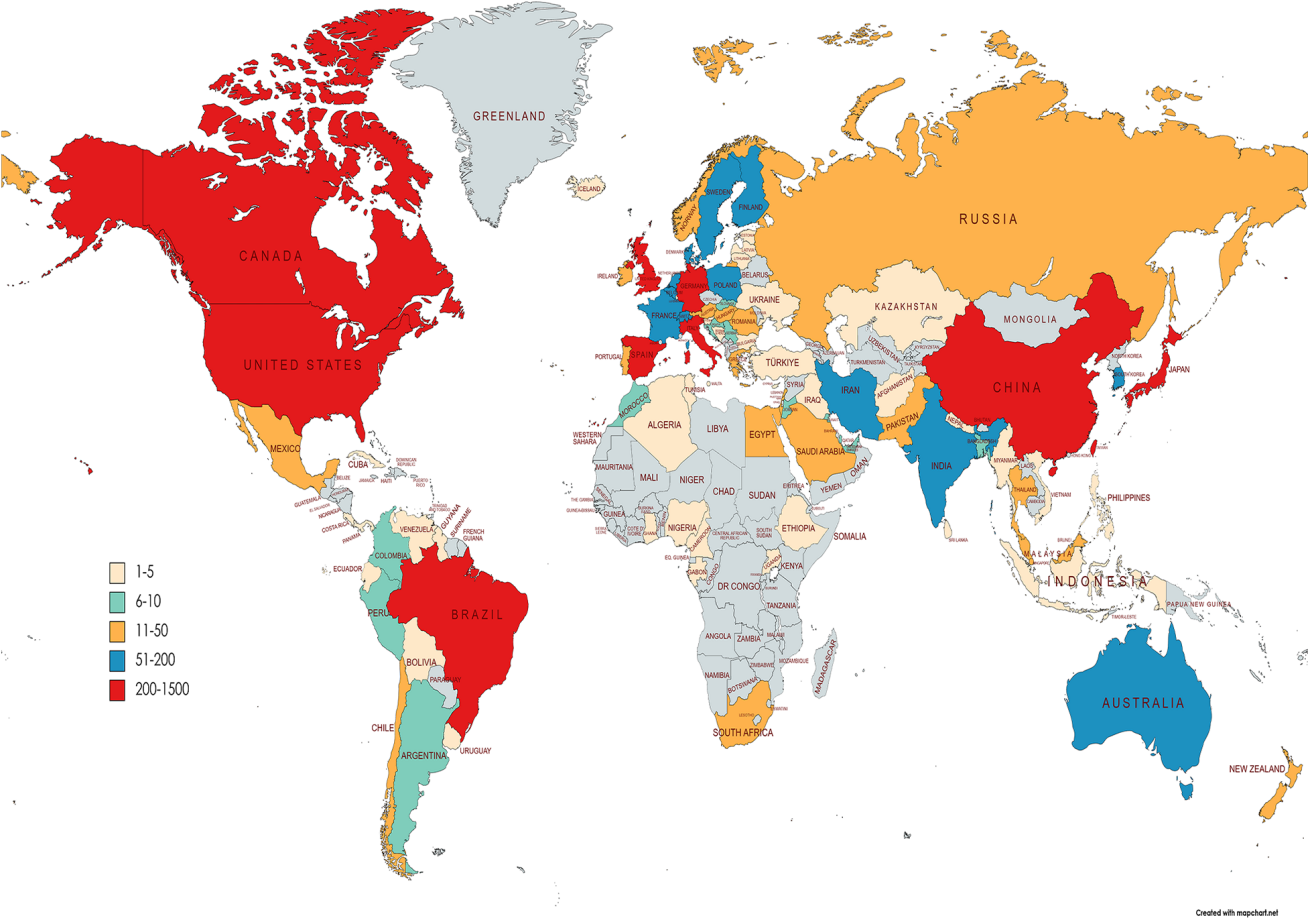
World map of total country output based on NP-related mood disorders.

**Figure 3:**
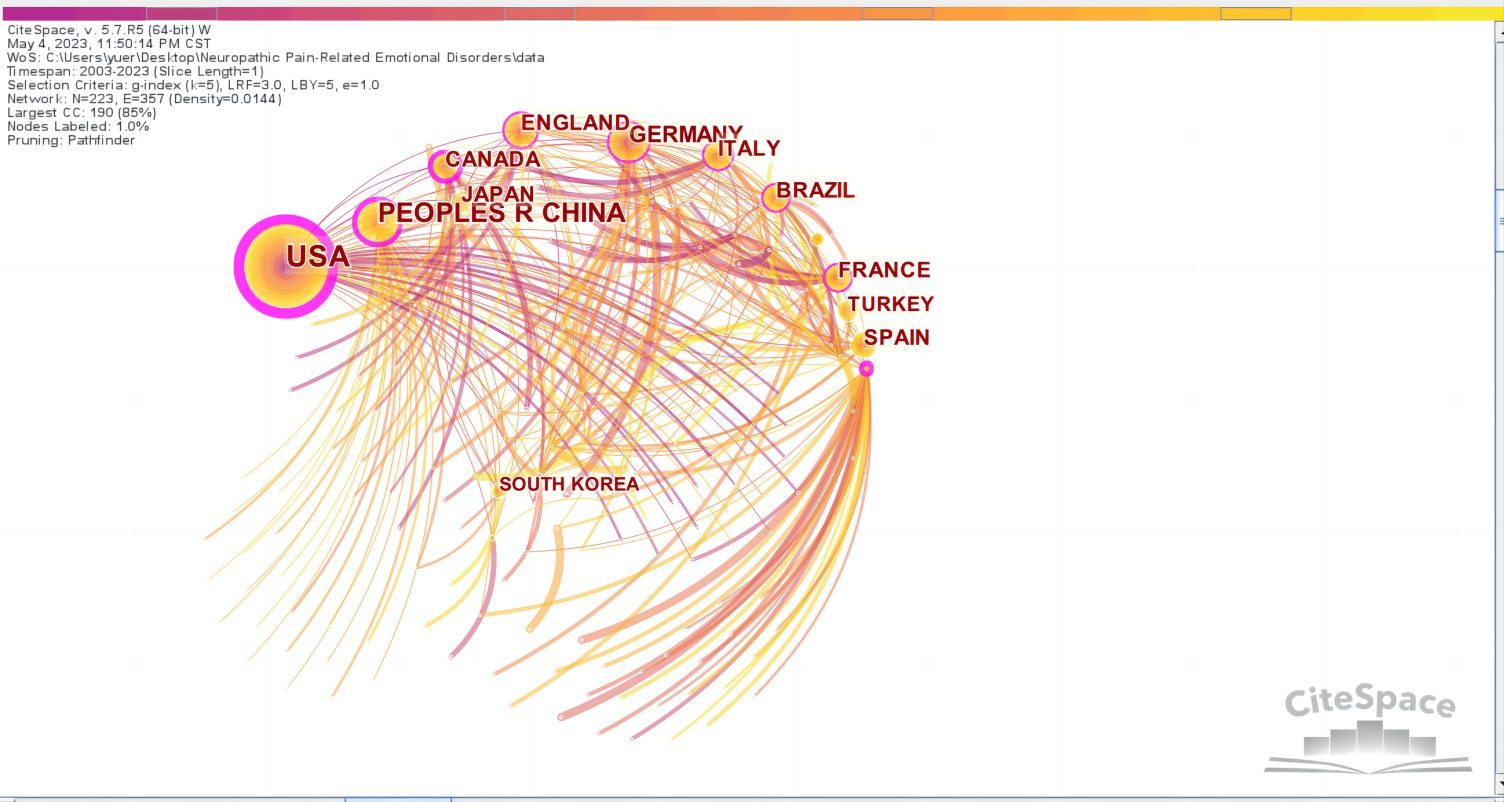
The collaboration network of countries related to NP-related mood disorders.

Through the quantitative analysis of the countries of publication, we can not only identify the core countries in the field of NP-related mood disorder research but also reflect on academic exchange and cooperation between countries in this field. Figure 2 shows a world map of all countries from which research on NP-related mood disorders has been published; the geographical distribution of publications covered 95 countries. After the country visualization analysis, 223 nodes and 357 lines were generated on the map (Figure 3).

Table 1 lists the top 10 countries in terms of frequency and centrality. Frequency represents the occurrence publications from a country, and centrality represents the position of a country in the field. As can be seen from the frequencies in Table 1, the Unites States of America (USA; 994) was the country with the highest number of articles in this field, far more than other countries. The USA had the highest centrality at 0.61, suggesting that most countries in the co-occurrence network had direct and indirect collaborations with the USA. Therefore, it is clear that the USA has played a vital role in this field. In addition, China (501) was second in the number of publications and ranked third in centrality (0.31), indicating that Chinese scholars have been actively participating in related research. Although Australia published only 74 articles, its centrality reached 0.37, ranking second, indicating that it was also in the core position in this field. However, less international collaborations were observed between countries and global cooperation has not been formed.

**Table 1:** Top 10 frequency and centrality of countries.

Rank	Frequency	Country	Year	Rank	Centrality	Country	Year
1	994	USA	2003	1	0.61	USA	2003
2	501	Peoples R China	2009	2	0.37	Australia	2004
3	196	Germany	2004	3	0.31	Peoples R China	2009
4	173	Italy	2003	4	0.24	Canada	2006
5	172	Canada	2006	5	0.18	Germany	2004
6	165	England	2005	6	0.17	Brazil	2006
7	159	Spain	2007	7	0.16	England	2005
8	139	France	2004	8	0.13	France	2004
9	133	Brazil	2006	9	0.11	Italy	2003
10	128	Japan	2005	10	0.09	Denmark	2004

### 3.3. Analysis of institutions

**Figure 4:**
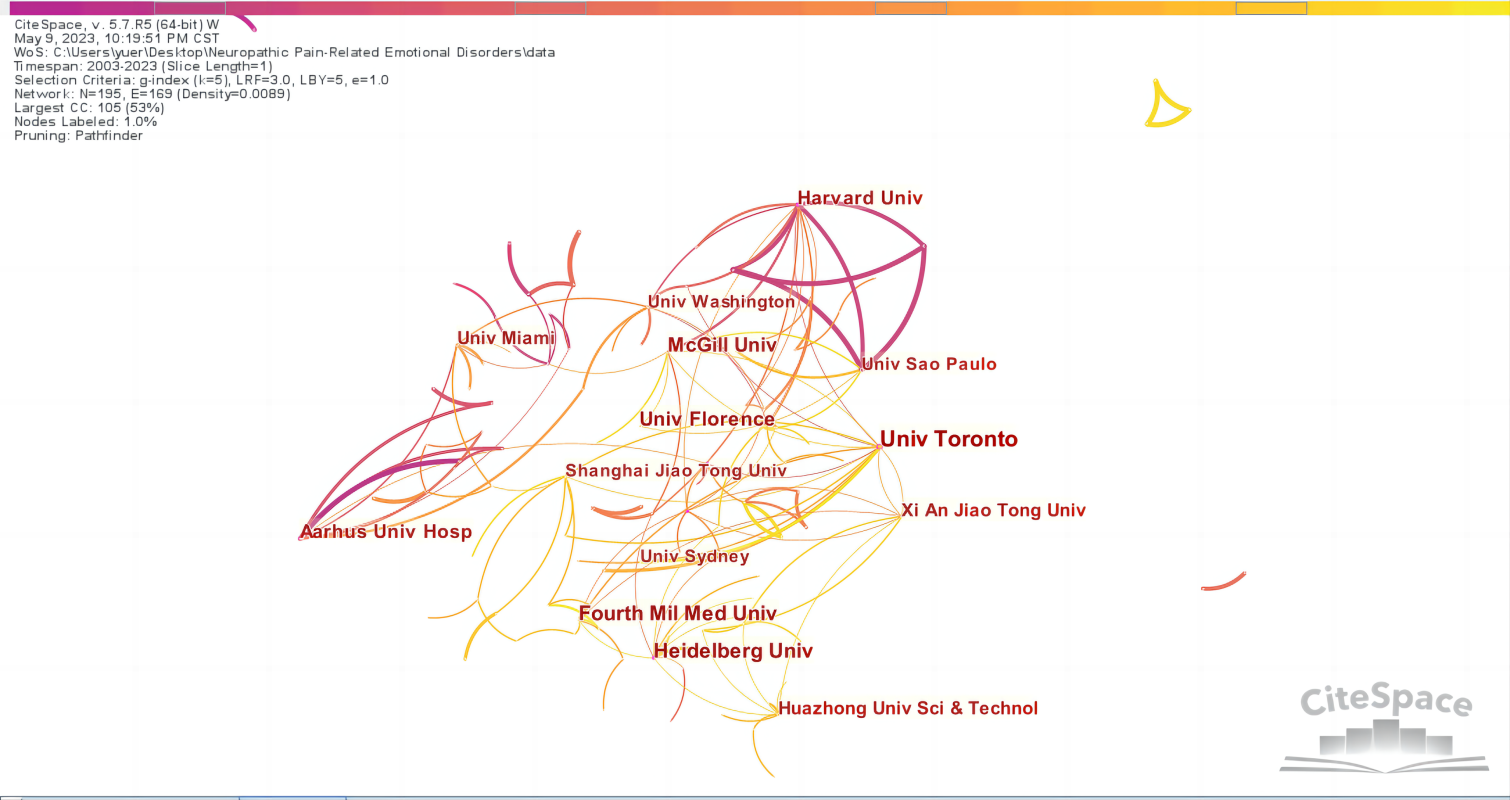
The collaboration network of institutions related to NP-related mood disorders.

By taking institutions as the analysis object in CiteSpace, an institutional analysis map with 195 network nodes, 169 connections, and a density of 0.0089 was obtained (Figure 4). Figure 4 shows that the nodes in the graph are relatively dense, and the data are in the majority, with a total number of 169 connections. Some of them have obvious regional characteristics. To promote academic exchanges, cross-agency research and cooperation need to be continued. At the same time, we summarized the top 10 institutions regarding frequency and centrality (Table 2). It can be seen that Univ Toronto (0.14), Heidelberg Univ (0.13), Harvard Univ (0.12), Karolinska Inst (0.11), and Aarhus Univ Hosp (0.09) were the leading institutional cooperative groups; the cooperation between these institutions was relatively close. The top five universities in the world were Univ Toronto (59), Heidelberg Univ (42), Fourth Mil Med Univ (41), Univ Florence (37), and McGill Univ (37). Univ Toronto not only had the highest number of publications (59) but also ranked first in terms of centrality (0.14), indicating that Univ Toronto is a core institution in the field and is in a leading position.

**Table 2:** Top 10 frequency and centrality of institution.

Rank	Frequency	Institution	Rank	Centrality	Institution
1	59	Univ Toronto	1	0.14	Univ Toronto
2	42	Heidelberg Univ	2	0.13	Heidelberg Univ
3	41	Fourth Mil Med Univ	3	0.12	Harvard Univ
4	37	Univ Florence	4	0.11	Karolinska Inst
5	37	McGill Univ	5	0.09	Aarhus Univ Hosp
6	33	Harvard Univ	6	0.09	Shanghai Jiao Tong Univ
7	29	Aarhus Univ Hosp	7	0.08	McGill Univ
8	27	Xi An Jiao Tong Univ	8	0.08	Johns Hopkins Univ
9	27	Huazhong Univ Sci & Technol	9	0.07	Fourth Mil Med Univ
10	26	Univ Miami	10	0.07	Pfizer Inc

### 3.4. Analysis of authors and cited authors

**Figure 5:**
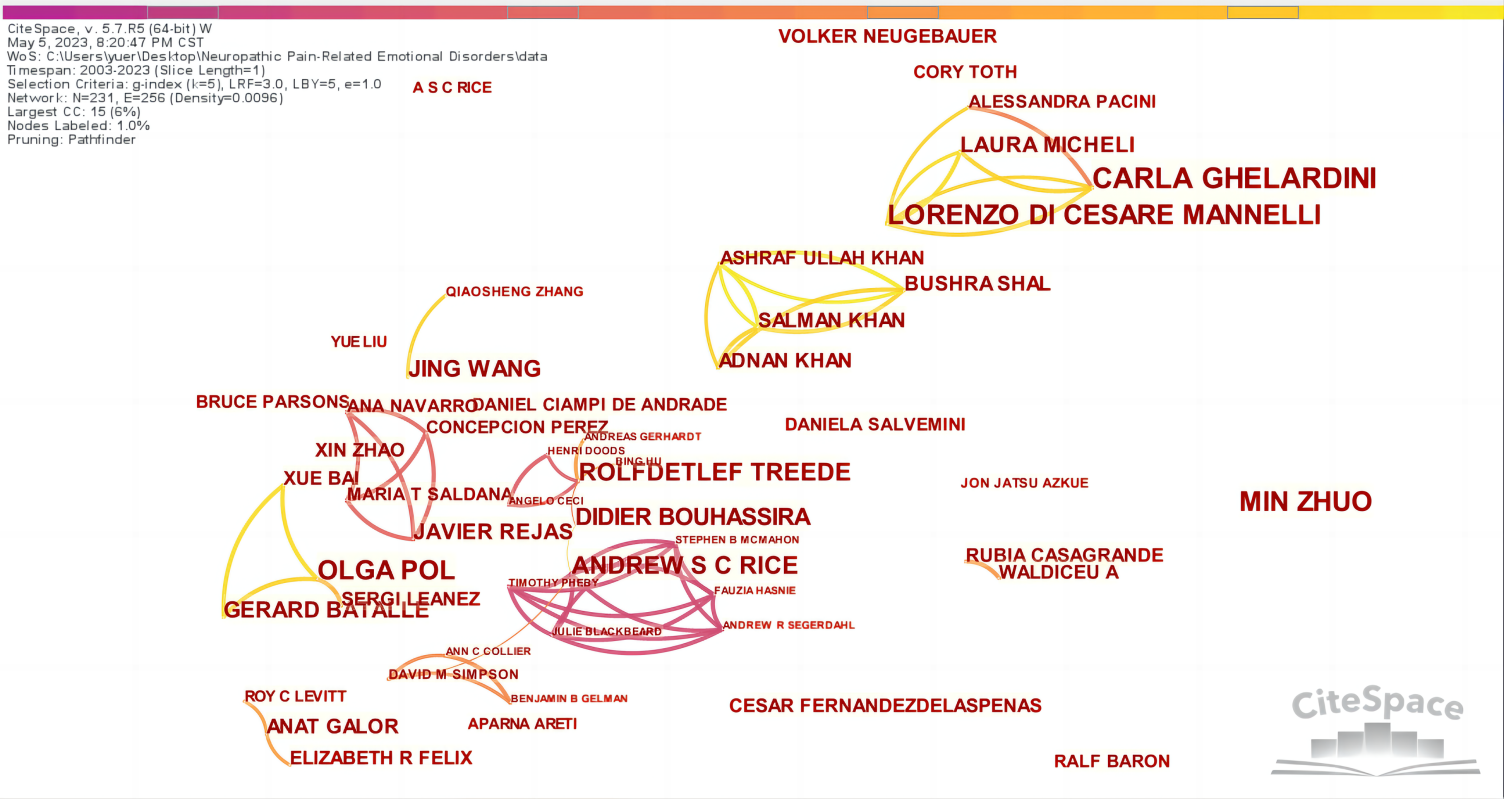
The collaboration network of authors related to NP-related mood disorders.

Through the visual analysis of authors in CiteSpace, Figure 5 was obtained, which is composed of 231 nodes and 256 lines, indicating a total of 231 authors, 256 instances of cooperation, and the formation connections between authors. The lines between nodes represent cooperative relationships (21). The visual atlas can visually display the information of influential research groups and potential collaborators and guide the establishment of collaborative relationships among researchers. Supplementary Table 1 shows that the top five authors were C GHELARDINI (20), M ZHUO (16), L DI CESARE MANNELLI (16), O POL (15), and ASC RICE (12). The core authors were determined according to Price's law, using the formula *M* = 0.749(*N*_max_)^1/2^, where N_max_ represents the maximum number of publications. Therefore, an N_max_ of 20 leads to an M of 3.349; rounding up, this is 4. Therefore, authors with at least four relevant papers were regarded as the core authors. There were 46 authors who published more than four articles. According to Price's law, when the number of papers published by core authors in a field reaches more than 50% of the total number of papers published in the field, it can be determined that a relatively stable core author group has been formed. The number of papers published by these 46 core authors (318) accounted for no more than 50% of the sample literature, indicating that a stable core author group had not been formed. This indicated that the existing high-yield academic team lacks cohesion, the number of core authors still needs to be increased, and the output efficiency of high-level articles needs to be improved.

**Figure 6:**
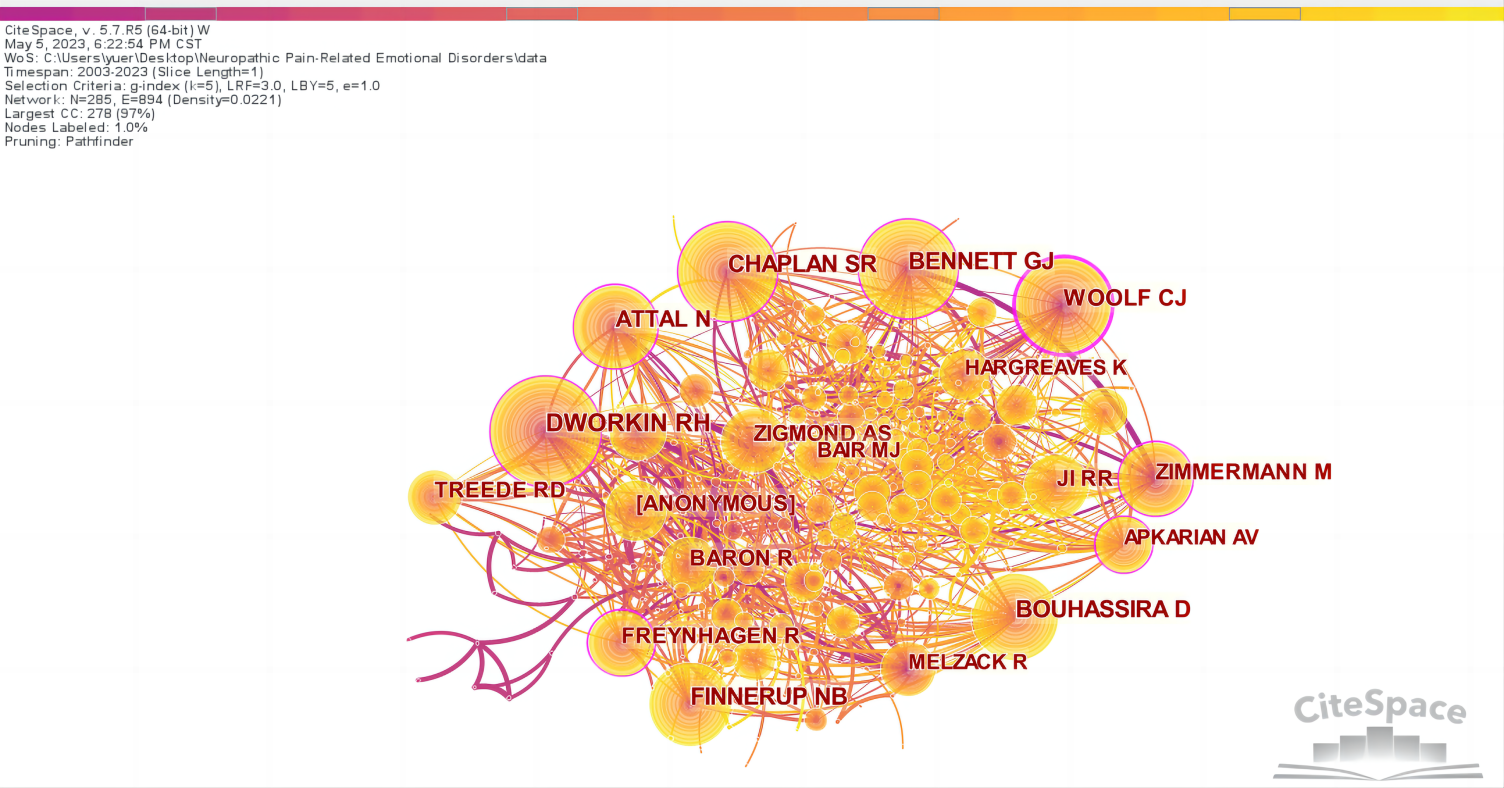
Map of cited authors related to NP-related mood disorders.

According to the co-citation analysis chart (Figure 6), there are 285 nodes and 894 lines. Supplementary Table 2 shows that the top five authors regarding citations were RH DWORKIN (411), SR CHAPLAN (403), GJ BENNETT (338), NB FINNERUP (323), and D BOUHASSIRA (320). The top five cited authors in terms of centrality were CJ WOOLF (0.29), RH DWORKIN (0.20), SR CHAPLAN (0.17), N ATTAL (0.17), and GJ BENNETT (0.13). Moreover, publications by GJ BENNETT and AM ZIMMERM were closely related.

### 3.5. Analysis of journals and cited journals

**Figure 7:**
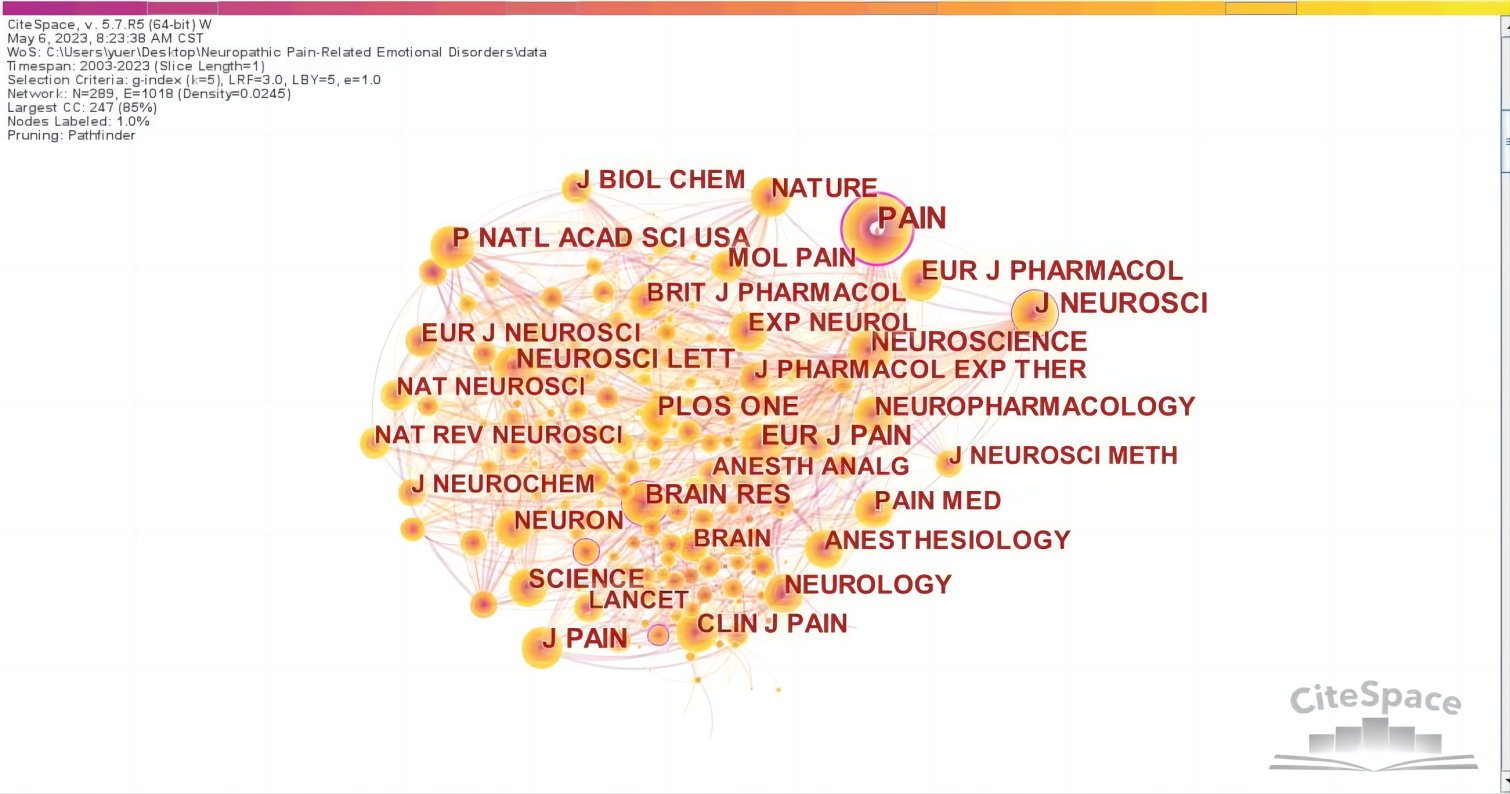
Map of cited journals related to NP-related mood disorders.

The top five journals with the most prolific research on NP-related mood disorders are shown in Table 3; these were *PAIN* (224), *EUROPEAN JOURNAL OF PAIN* (90), *PAIN MEDICINE* (82), *NEUROSCIENCE LETTERS* (68), and *INTERNATIONAL JOURNAL OF MOLECULAR SCIENCES* (66), with an average impact factor of 4.9238, which indicates that there is a certain proportion of high-quality articles in this field. In addition, the cited journals were mapped (Figure 7), yielding 289 nodes and 1,018 lines. Nodes in the map represent journals, and the lines between nodes represent co-citation relationships. The top 10 most cited journals are shown in Supplementary Table 3; *PAIN* had the highest number of citations (2,912) and centrality (0.34) and was the most authoritative journal in the field. The top 10 journals were all cited more than 1,000 times.

**Table 3:** Top 5 most productive journals.

Rank	Publication	Journal	Impact factor (2022)	Quartile
1	224	Pain	7.926	Q1
2	90	European Journal of Pain	3.651	Q2
3	82	Pain Medicine	3.637	Q2
4	68	Neuroscience Letters	3.197	Q3
5	66	International Journal of Molecular Sciences	6.208	Q1

**Figure 8:**
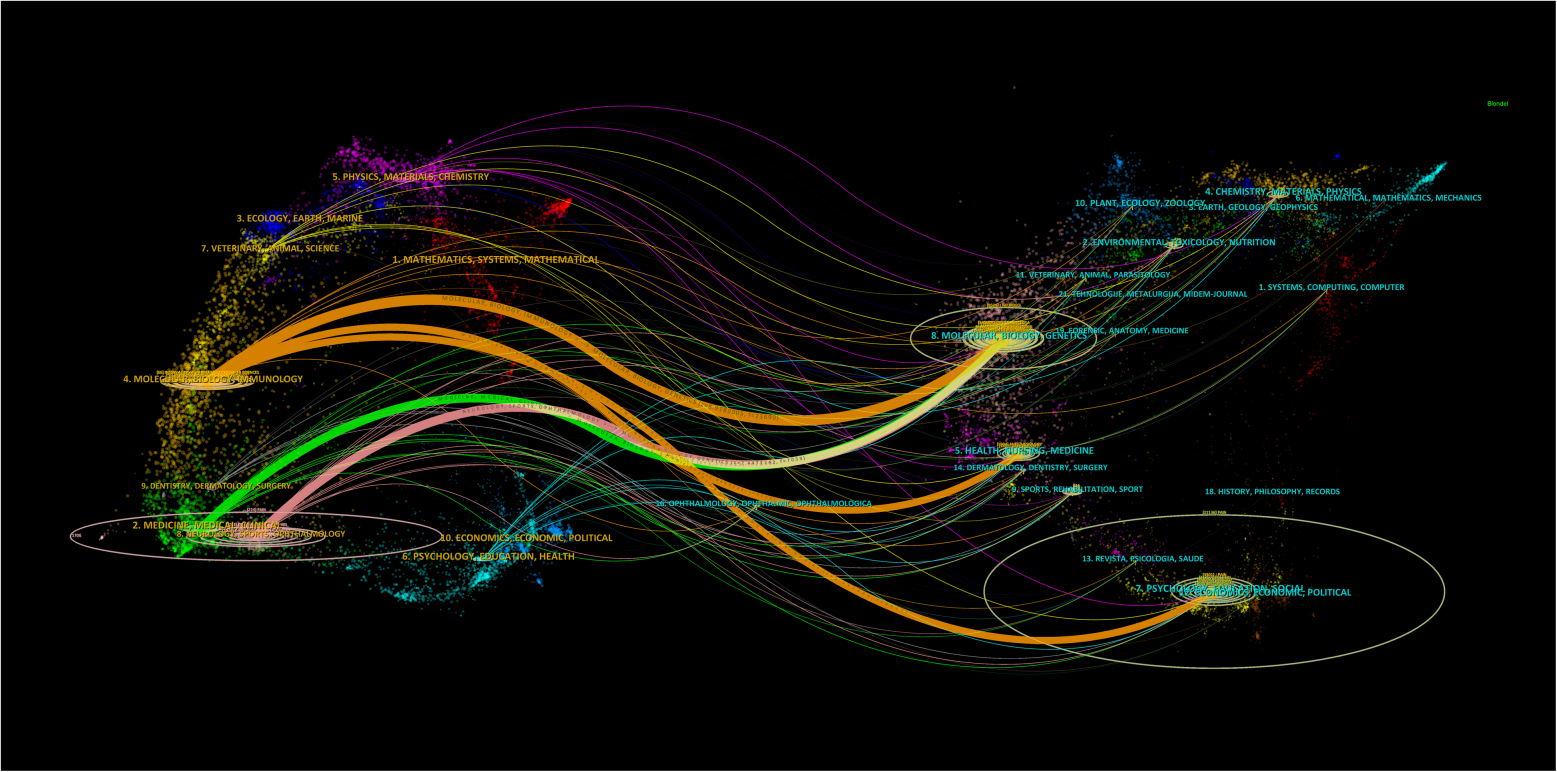
The dual-map overlay of journals related to NP-related mood disorders.

Citation analysis is the most commonly used method to identify the structure and dynamic evolution of professional knowledge (22). From the perspective of bibliometrics, citation forms the research frontier, and the cited literature forms the knowledge base. The dual-map overlay map of the subject distribution involved in a research field can be obtained by superimposing the information service data on the original subject base map of the citation-cited literature using Addoverlay (Figure 8). The left side of Figure 8 shows the subject distribution of cited literature as the research status quo; the right side is based on the discipline to which the cited literature belongs. The wavy curve is the citation line, which completely shows the context of the citation. The numbers inside the ellipses represent the number of papers published by each discipline; the length of the ellipse represents the number of authors, and the width of the ellipse represents the number of publications. The z-scores function highlights stronger and smoother trajectories, with higher values of the function indicating thicker lines and stronger effects. As shown in Figure 8, citations of “molecular, biology, immunology” (orange lines) were significantly affected by “molecular, biology, genetics” (*z* = 8.9189005, *f* = 23,690), “health, nursing, medicine” (*z* = 1.8480134, *f* = 5,519), and “psychology, education, social” (*z* = 2.127409, *f* = 6,237); “medicine, medical, and clinical” (green lines) were significantly influenced by “molecular, biology, and genetics” (*z* = 2.559733, *f* = 73,481).

### 3.6. Analysis of cited references

**Figure 9:**
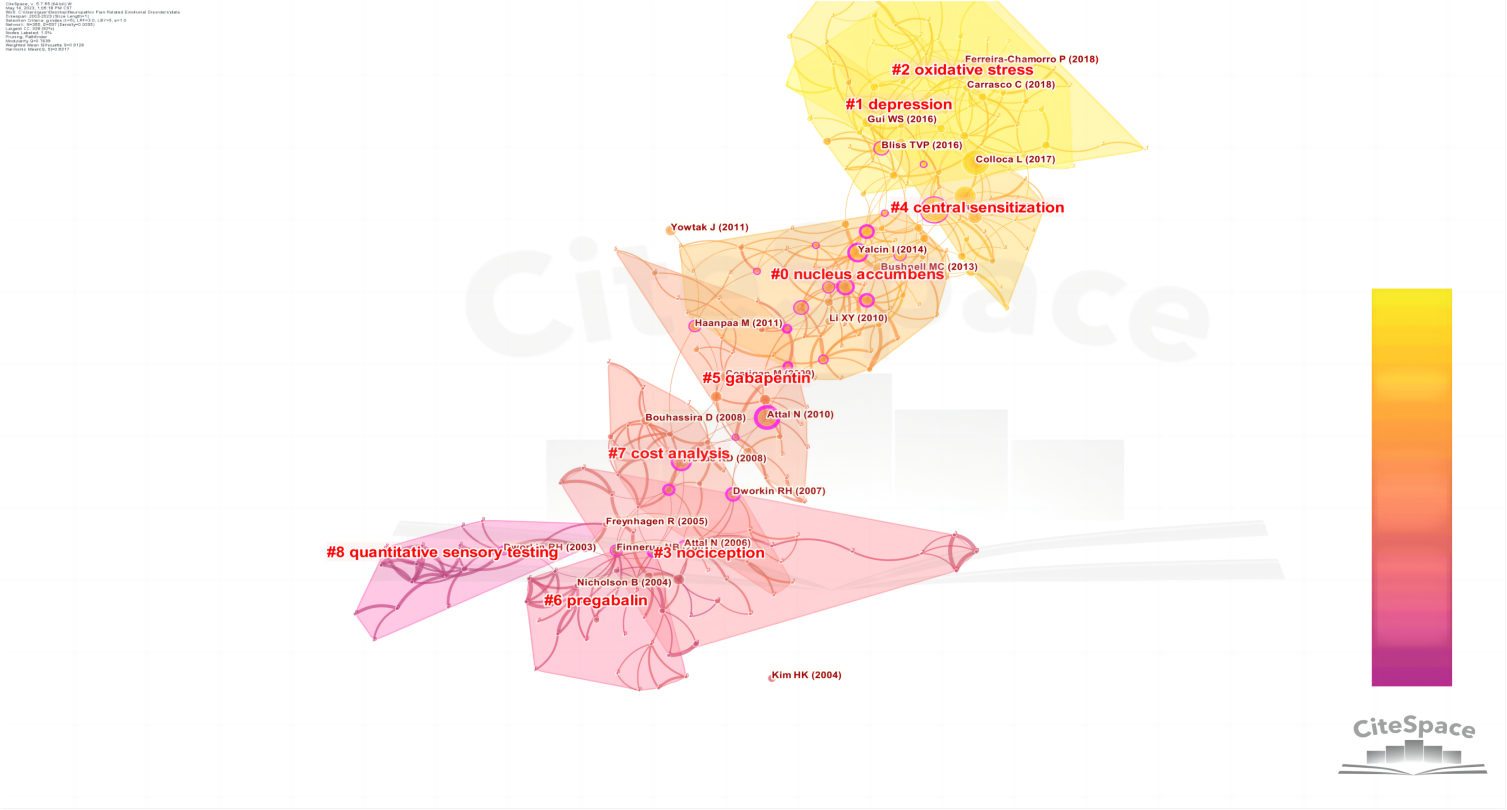
The cluster map of cited references related to NP-related mood disorders.

Literature co-citation analysis can identify key studies and research frontiers in a field. Key studies play an important role in laying the foundation and promoting the formation and development of a discipline or research field. A co-citation map of cited references is shown in Supplementary Figure 2, and it contained 355 nodes and 597 lines. The top 10 cited articles (Supplementary Table 4) were all from the Q1 region, and the average impact factor was as high as 26 points, indicating that there were a large number of high-quality papers in this field. In terms of the citation frequency, the top five articles were published by L COLLOCA in 2017 (72), NB FINNERUP in 2015 (65), N ATTAL in 2010 (41), RD TREEDE in 2008 (39), and MC BUSHNELL in 2013 (38). It can be seen that I YACLIN (2014), C ALBA-DELGADO (2013), and I YALCIN (2011) were closely related, indicating that these references were often cited together. L COLLOCA (2017) and NB FINNERUP (2015, 2016) had a high level of co-citation, which indicates that these studies are also highly similar in content. Figure 9 displays a cluster map of the cited references. The Q value was 0.7639 and the S value was 0.9128, indicating a large internal structure and providing evidence for the validity and reliability of the clustering results. The top 9 clusters were “nucleus accumbens”, “depression”, “oxidative stress”, “nociception”, “central sensitization”, “gabapentin”, “pregabalin”, “cost analysis”, and “quantitative sensory testing”.

### 3.7. Analysis of keywords

**Figure 10:**
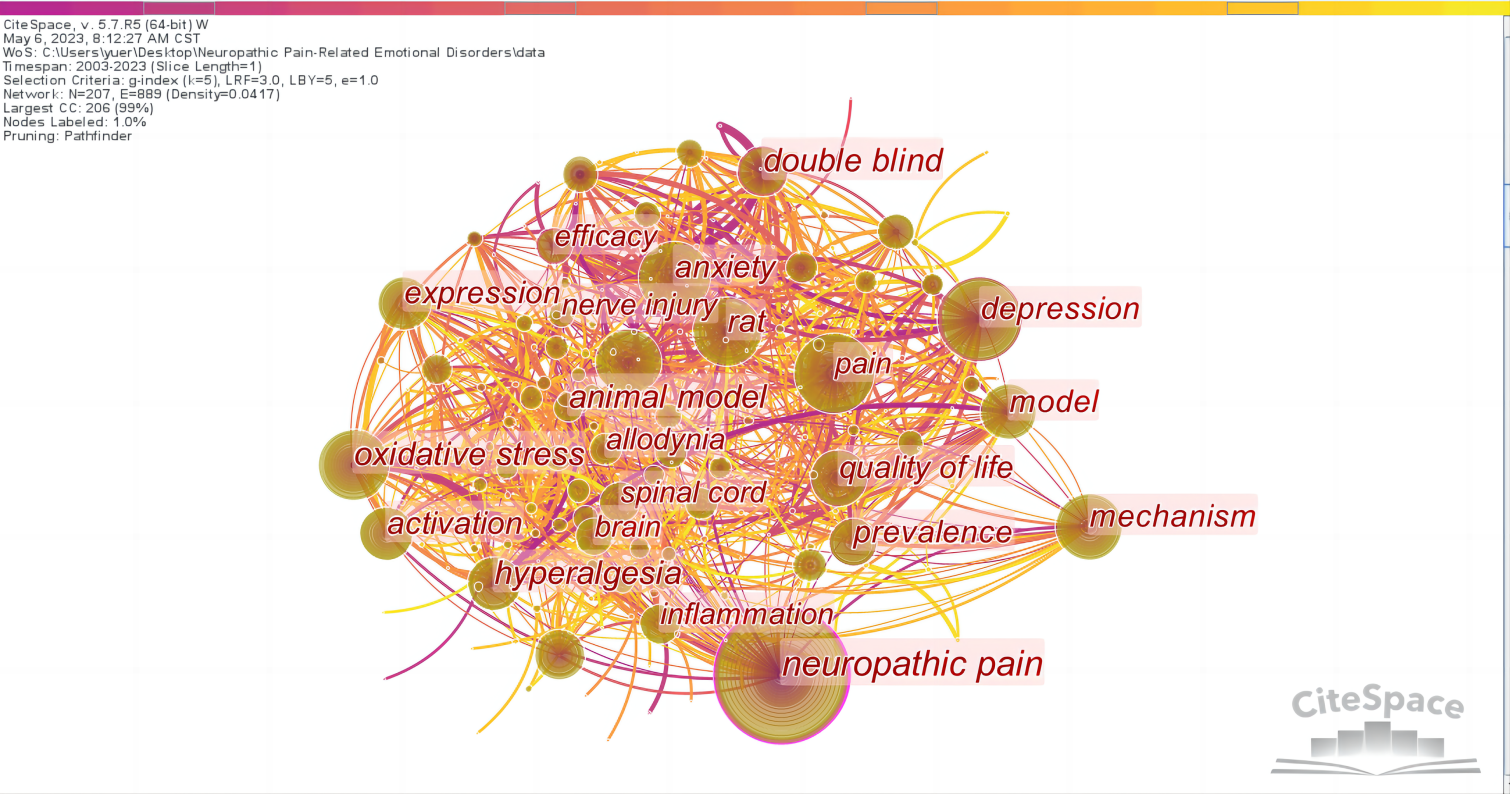
Map of keywords related to NP-related mood disorders.

The collection of keywords given in a large number of academic studies in a research field can reveal the overall content characteristics of the research results, the internal relationship between the research contents, and the development and directions of the research (23). Keyword analysis is used to understand the essence of a certain field and identify current research hotspots and future directions (24). A network map of keywords was generated, consisting of 207 nodes and 889 links (Figure 10). According to the frequency and centrality of keywords (Supplementary Table 5), the hot keywords were “neuropathic pain”, “depression”, “pain”, “oxidative stress”, “anxiety”, “mechanism”, “rat”, “activation”, “chronic pain”, “model”, “quality of life”, “double blind”, “prevalence”, and “efficacy”.

**Figure 11:**
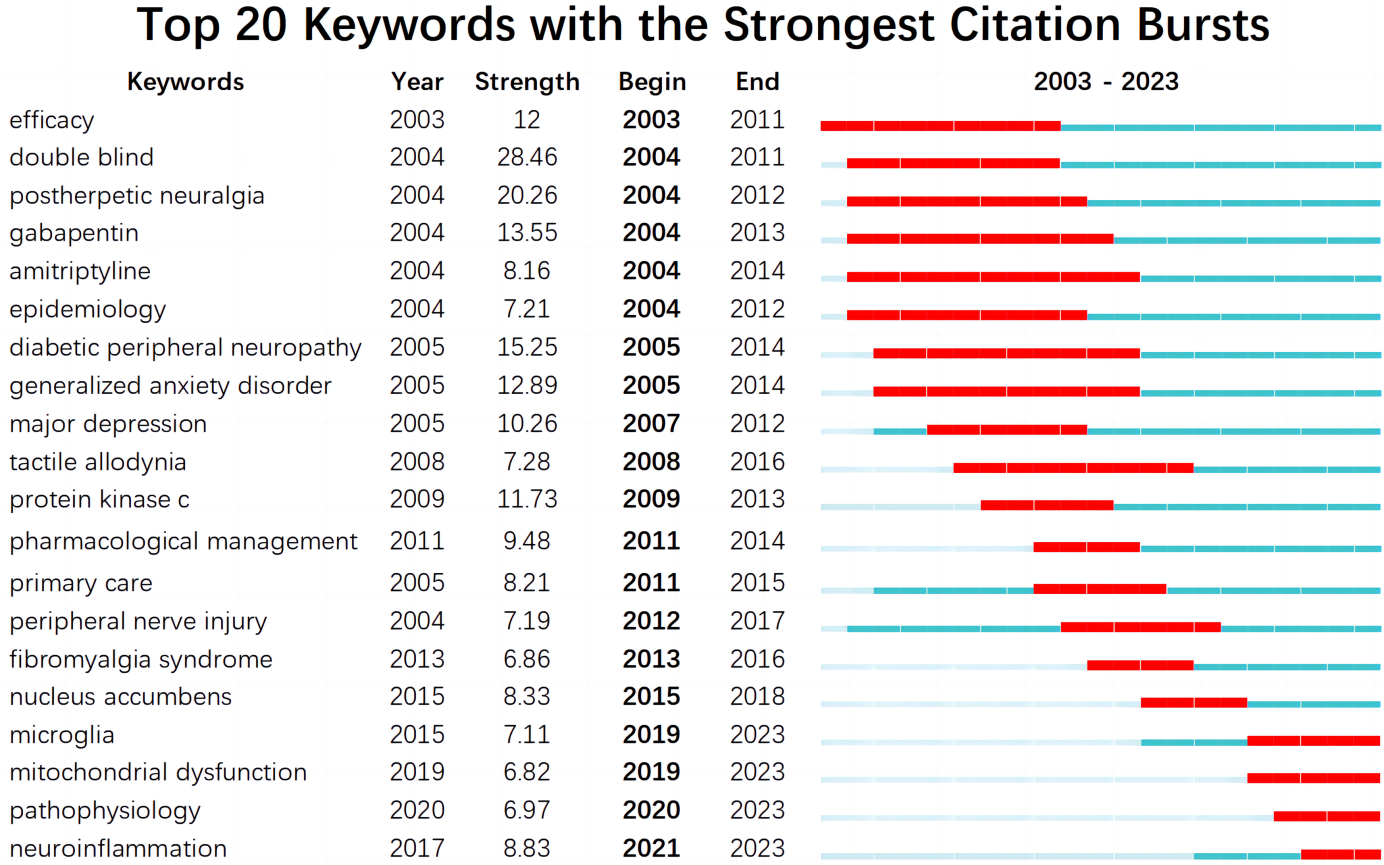
Top 20 keywords with the strongest citation bursts.

CiteSpace is specifically designed to help detect new trends and sudden changes in the scientific literature. In CiteSpace, current research frontiers are identified based on “burst words” extracted from titles, abstracts, descriptors, and identifiers of bibliographic records (25). Burst words are words that suddenly appear in large numbers, which show turning points or new discoveries in the development of a discipline. These are likely to become research hotspots in the future and lead the development of a discipline. Through changes in research hotspots over time, the process of development and trends in a field can be seen, allowing further predictions of keywords that can help predict the future explosive trends. The top 20 keywords with the strongest bursting were obtained by a time series burst detection of the highly cited keywords (Figure 11). The time interval of emergence is indicated by the blue line. The time period in which bursts were found for a subject category is shown as red line segments, indicating the start and end years of the burst (17). As can be seen from Figure 11, the keywords with the maximum and minimum emergence intensity were “double blind (28.46)” and “mitochondrial dysfunction (6.82)”, respectively, and the average intensity value was 10.92. The hot keywords were “postherpetic neuralgia”, “diabetic peripheral neuropathy”, “gabapentin”, “generalized anxiety disorder”, “efficacy”, “major depressive disorder”, and “protein kinase c”; the emergence intensity of these seven keywords all exceeded 10, second only to “double blind”. The most recent burst keywords were “microglia”, “mitochondrial dysfunction”, “pathophysiology”, and “neuroinflammation”. There was a correlation between the above keywords, and these keywords were also the research frontiers in NP-related mood disorder research.

Performing a cluster analysis and summarizing these keywords can offer a more comprehensible comprehension of the current research topics related to NP-related mood disorders (Supplementary Figure 3). After clustering, the Q value was 0.3983 and the S value was 0.7143, indicating that clustering was efficient and meaningful. A total of six clusters were generated to reflect current trends, and these were “quality of life”, “oxidative stress”, “pregabalin”, “microglia”, “spinal cord”, and “hippocampus”.

**Figure 12:**
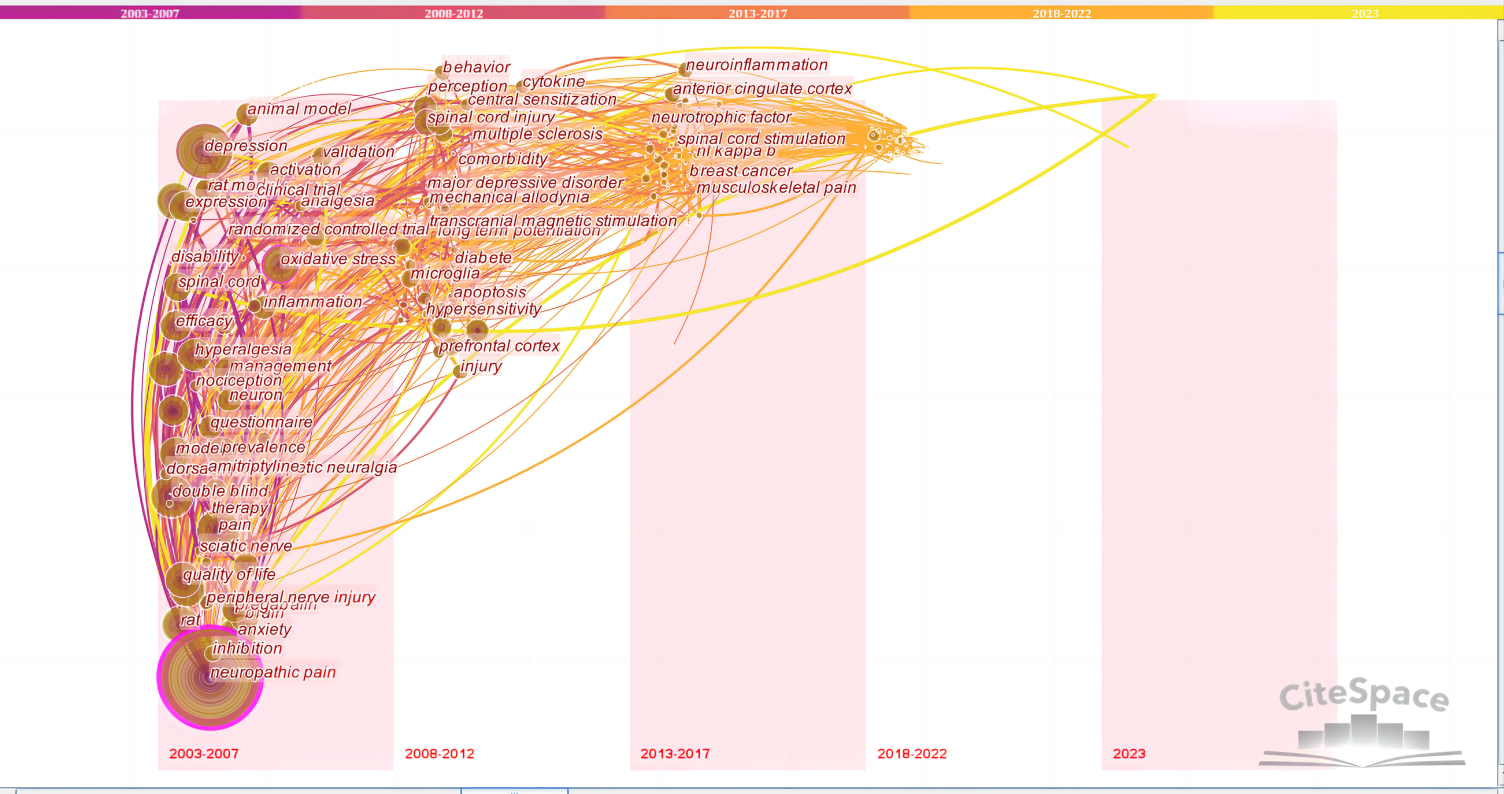
The timezone view of keywords related to NP-related mood disorders.

The timezone view provides another map that focuses on the evolutionary trends of keywords across time, which can clearly show updates and the mutual influence of studies on each other. Each time period in the timezone map shows the newly appearing keywords in that time period. If they co-appeared with previous keywords in the same article, they were linked with a line. The previous keyword frequency then increased by 1, and the circle became larger. From the timezone view (Figure 12), it can be seen that the early studies were mainly clinical studies on “quality of life”, “double blind”, “efficacy”, and “prevalence”. Common keywords then gradually changed to “oxidative stress”, “neuroinflammation”, “microglia”, “neurotrophic factor”, and “anterior cingulate cortex”, among mechanism studies.

## 4. Discussion

Pain is a complex physiological and psychological process and is one of the most common clinical symptoms. The International Association for the Study of Pain defines neuropathic pain as “pain caused by a lesion or disease of the somatosensory nervous system” (26). NP is often accompanied by anxiety, depression, and other mood disorders, affecting approximately 34% of NP patients and significantly impacting their quality of life. Unfortunately, despite the prevalence and severity of these comorbid conditions, there is currently a lack of effective and safe treatments to address both the symptoms of NP and its associated mood disorders (27). NP is a chronic pain state caused by peripheral or central nerve injury or systemic disease. In clinical practice, the comorbidity of chronic NP and anxiety/depression is quite common. Depression is a prevalent mood disorder that manifests a diverse range of symptoms, impacting somatic, cognitive, affective, and social processes across the lifespan, including adolescence, midlife, and the elderly. Anxiety is a psychological state characterized by a sense of apprehension, unease, or distress that arises in response to perceived threats or stressors. Physical pain and emotional pain are intertwined and aggravate each other, which increases the disability rate, lengthens the course of disease, worsens prognosis, makes treatments more complex and difficult, and increases medical burden.

According to the literature co-citation cluster map (Figure 9), the words with high clustering strength were “nucleus accumbens”, “depression”, “oxidative stress”, “nociception”, “central sensitization”, “gabapentin”, “pregabalin”, “cost analysis”, and “quantitative sensory testing”. According to the keyword co-occurrence map (Figure 10), the research topics on NP-related mood disorders with high frequency and centrality mainly included “oxidative stress”, “mechanism”, “activation”, “quality of life”, “double blind”, “prevalence”, and “efficacy”. According to the keyword emergence map (Figure 11), “double blind”, “postherpetic neuralgia”, “diabetic peripheral neuropathy”, “gabapentin”, and “efficacy” were the main keywords with high emergence intensities. In recent years, the frontier research areas have mainly focused on “microglia”, “mitochondrial dysfunction”, “pathophysiology”, and “neuroinflammation”. According to the keyword cluster map (Supplementary Figure 3), the cluster words mainly included “quality of life”, “oxidative stress”, “pregabalin”, “microglia”, “spinal cord”, and “hippocampus”. From the timezone map (Figure 12), we also found that “neurotrophic factors” and “ACC” (anterior cingulate cortex), with other brain areas, have become popular words in recent years. The analysis of these keywords can well identify the research hotspots and frontiers in this field.

### 4.1. Research hotspots

#### 4.1.1. “Neuropathic pain”, “depression”, “anxiety”, “chronic pain”, “quality of life”, “primary care”, “generalized anxiety disorder”, “major depressive disorder”, “cost analysis”, “quantitative sensory testing”

NP usually develops into a long-term chronic pain state, often accompanied by negative mood disorders such as anxiety and depression. Pain and negative emotions often form a vicious circle, seriously affecting quality of life and causing economic burden. The healthcare costs associated with NP-related mood disorders can be significant. This is due to the need for treatment of NP itself, as well as the additional challenges posed by co-existing mood disorders, requiring comprehensive medical and psychological support. Pain, anxiety, and depression are common in primary care patients, and there is considerable evidence that these experiences are correlated. One study investigated the associations between pain symptoms and symptoms and diagnoses of anxiety and depression in primary care patients. The results suggested that primary care patients with supportive muscle pain, headache, or stomach pain symptoms were approximately 2.5–10 times more likely to screen positive for panic disorder, generalized anxiety disorder, or major depressive disorder (28). Among primary care patients with chronic pain, nearly half screened positive for one or more anxiety disorders, which in turn were inversely associated with multiple domains of impairment in health-related quality of life. Detection and treatment of anxiety may be an important component of pain management (29). Quantitative sensory testing plays a significant role in NP by quantitatively assessing patients' sensory function and pain sensitivity, aiding in diagnosis and severity evaluation of the condition, and facilitating personalized treatment plans (30).

#### 4.1.2. “Pregabalin”, “gabapentin”, “amitriptyline”, “pharmacological management”

Pregabalin and gabapentin are calcium channel modulators, which were originally used as anticonvulsants for the treatment of epilepsy. At present, they are the first-line drugs for the clinical treatment of NP with excellent analgesic effects (31, 32). Studies have shown that pregabalin can effectively improve pain symptoms, sleep disorders, anxiety, and depression in patients with NP (33). In addition, gabapentin can relieve chronic NP and depression-like behaviors in rat models of NP and depression and can promote neurogenesis in hippocampal dentate gyrus neurons (34). Amitriptyline, as a tricyclic antidepressant, has analgesic, sedative, and antidepressant effects and is also widely recommended for the treatment of NP and related mood disorders (35). They are all commonly used in the pharmacological management of NP and related mood disorders. These medications can play a role in alleviating pain symptoms and managing associated mood symptoms.

#### 4.1.3. “Postherpetic neuralgia”, “diabetic peripheral neuropathy”

Postherpetic neuralgia (PHN) is a severe and refractory chronic neuralgia syndrome after the healing of herpes zoster lesions. PHN typically develops within weeks after the shingles rash has healed. Its primary symptom is severe pain in the affected area, often described as burning or stabbing. The pain can persist for months or even years, significantly impacting the patient's quality of life. In some cases, PHN can lead to sleep disturbances, anxiety, depression, and limitations in daily activities.Painful diabetic peripheral neuropathy (DPN) is the most common complication of diabetes mellitus, which can easily cause diabetic peripheral neuralgia. DPN is caused by damage to the nerves due to high blood sugar levels and prolonged exposure to uncontrolled diabetes. Over time, the elevated blood glucose levels can lead to nerve damage and impaired nerve function, resulting in abnormal sensations and pain. PHN and DPN are commonly studied peripheral neuropathic pain syndromes. Although the etiology of the two is different, they have a common pathogenesis; that is, they are caused by sensitization of the peripheral and central nervous systems (36). For patients with these conditions, alleviating pain and improving quality of life are important treatment goals.

#### 4.1.4. “Central sensitization”, “tactile allodynia”, “nociception”, “plasticity”

Central sensitization, a form of pain hypersensitivity, is attributed to central neural plasticity and is intricately linked to psychoneuroimmunological interactions. This phenomenon holds significant relevance in the realm of pain diagnosis and treatment (37). Central sensitization is typified by hyperalgesia, specifically dynamic tactile hyperalgesia, secondary punctate or compressive hyperalgesia, and augmented aftersensation and temporal summation. It can be elicited easily and rapidly by applying a variety of experimental nociceptive conditioning stimuli to the skin, muscle, or viscera and, in addition to producing hyperalgesia, leads to secondary changes in brain activity that can be detected by electrophysiological or imaging techniques (38). Synaptic plasticity refers to the ability of synapses, the connections between neurons, to change their strength and efficacy based on patterns and frequencies of neural activity. The central sensitization caused by functional and structural synaptic plasticity in the posterior horn of the spinal cord is considered to be the common pathological basis of this variety of NP. Functional synaptic plasticity is characterized by the weakening of inhibitory synapses and strengthening of excitatory synapses, and structural plasticity is characterized by an increase in the number and size of dendritic spines in excitatory synaptic regions. Previous studies have shown that T-cell lymphoma invasion and metastasis-inducing protein 1 (Tiam1) is a key factor in the pathophysiology of chronic pain-induced depressive-like behaviors and the sustained antidepressant-like effects of ketamine (39). Recent studies have shown that Tiam1–Rac1 signaling in excitatory neurons in the posterior horn of the spinal cord is involved in the occurrence, transformation, and maintenance of a variety of NP types by coordinating structural and functional synaptic plasticity (40).

#### 4.1.5. “Double blind”, “efficacy”, “prevalence”, “randomized controlled trial”

Double blinding is a method in study design that aims to reduce subjective bias, while randomized controlled trials use a study design that evaluates the effects of treatment by randomly assigning participants to different groups. Double blinding is often used in randomized controlled trials to ensure the objectivity and reliability of research. In terms of clinical research, the research in NP-related mood disorders was mainly based on the simple evaluation of clinical efficacy and prevalence surveys at first, and then the number of high-quality double-blind clinical trials and randomized controlled trials gradually increased. Initially, research in this field primarily focused on describing and understanding the clinical manifestations and comorbidities of NP-related mood disorders. However, there has been a shift towards investigating the underlying mechanisms that contribute to the development and maintenance of these mood disorders. Researchers are now exploring various aspects related to NP and mood disorders, including neurobiological, genetic, and psychosocial factors. They aim to uncover the intricate mechanisms that connect NP with alterations in mood and emotional regulation. In terms of basic research, research in this field has begun to focus on immune and inflammatory mechanisms.

#### 4.1.6. “Rat”, “model”, “hippocampus”, “BDNF”

Using rats as an animal model, basic research on NP-related mood disorders has increased greatly (41, 42). Studies in recent years have focused on brain-derived neurotrophic factor (BDNF) as a hot topic. BDNF is a member of the neurotrophic factor family, functioning as a protein that significantly influences the development, differentiation, growth, regeneration, and function of nerve cells. An increasing number of investigations have demonstrated the involvement of BDNF and its highly binding receptors, which are widely distributed in central and peripheral neurons, in the genesis and persistence of pain (43, 44). BDNF is mainly expressed in pyramidal cells and granule cells in the CA1 and CA2 regions of the forebrain and hippocampal dentate gyrus, and it is closely related to the regulation of hippocampal neurogenesis (45). In NP, hippocampal BDNF deficiency has been widely reported and is thought to be associated with cognitive and depressive symptoms (46). Animal models have shown that, as a marker of neuroplasticity, BDNF modifies the structure and function of the hippocampus (41). In addition, BDNF may be an effective target for the treatment of comorbid diabetic NP and depression (47).

### 4.2. Research frontiers

#### 4.2.1. “Microglia”, “activation”, “oxidative stress”, “mitochondrial dysfunction”, “pathophysiology”, “neuroinflammation”

Over the past two decades, neuroimmunity has been an active field in pain research. Numerous studies have shown that interactions between the nervous and immune systems play key roles in the establishment and maintenance of NP. However, studies have found that neuroimmunity is also involved in pain relief. NP may be related to the dysfunction of immune cells and their cytokines involved in pain relief (48). Oxidative stress, mitochondrial dysfunction, and neuroinflammation are the main pathological mechanisms that trigger NP (37, 49). The term “oxidative stress” denotes the overproduction of reactive oxygen species within cells and tissues, coupled with the incapacity of the antioxidant system to counterbalance the disparity between the production of reactive oxygen species and their removal by protective mechanisms, potentially resulting in persistent inflammation. Oxidative stress has the potential to activate a diverse range of transcription factors, which may cause the selective expression of certain genes implicated in inflammatory pathways, thereby instigating chronic pain (50). In addition, in the nervous system, increased oxidative stress causes neuronal apoptosis, and studies have shown that inhibiting neuronal apoptosis can alleviate NP symptoms (51). As a source of cellular reactive oxygen species, mitochondria have regulatory effects on cellular oxidative stress and subsequent inflammatory signal transduction. Mitochondrial dysfunction is also an important mechanism of the occurrence and development of NP, which can induce depression, anxiety, and other mood disorders (52, 53). Ferroptosis is a newly identified mechanism of cellular demise that is distinguished by mitochondrial impairment, oxidative stress, and mitochondrial dysfunction, which selectively impacts certain forms of synaptic plasticity in the spinal cord. Results showed that ferroptosis is involved in the development of NP in male rats by blocking the activation of neurons and astrocytes in the spinal dorsal horn (54). Keyword analysis suggested that “microglia” was a hot keyword in this field. As an important immune cell in the brain, microglia maintain the homeostasis of the central nervous system. In the process of pain recognition, multiple brain regions are activated at the same time, and abnormally activated microglia release inflammatory mediators, cause neuroinflammation, and enhance long-term harmful nerve signal transmission, leading to central pain sensitization, leading to and aggravating NP, and participating in the formation and maintenance of anxiety and depression (55, 56). Microglial activation was found to attenuate synaptic transmission and reduce neuroinflammation, synaptic function, and neuralgia. In rats with spared nerve injury, NP and depression-like behavior coexist, and activated microglia and inflammatory factors are increased in the medial prefrontal cortex (mPFC), indicating that microglial activation is closely related to NP and depression (42). Researchers have also found the presence of hippocampal microglia senescence in spared nerve injury mice, and microglia showed inflammation-related aging markers (57). Another study suggested that hippocampal microglial activation is an important mechanism for anxiety and depression secondary to chronic NP (58). Activated microglia release inflammatory mediators and cytokines in neuropathic pain, such as tumor necrosis factor *α* and interleukin-1β, which stimulate neurons to produce pain signals. At the same time, the activation of microglia can also trigger oxidative stress responses, leading to oxidative damage and the aggravation of neuroinflammation, further increasing the occurrence and development of NP, and affecting the emotional regulation system, leading to the appearance of emotional disorders related to NP. Microglia activation, oxidative stress, and neuroinflammation are closely related to neuropathic pain-related mood disorders. In the future, in-depth research on the mechanisms behind the involvement of microglia in NP and mood disorders will help deepen the understanding of the pathogenesis of NP and provide a theoretical basis for the development of new treatment strategies.

#### 4.2.2. NP-related mood disorders related to brain areas

An important role played by the brain is in the central regulation of pain as well as in the generation of negative emotions (59). Current research on pain and emotions has focused on the central nervous system, pain emotion-related nuclei, and the connections between them. Neurons connect and transmit information through synapses, forming neural circuits. The most common synaptic transmission is neurochemical; that is, presynaptic neurons release neurotransmitters that bind with the corresponding receptors in postsynaptic membranes, resulting in changes in membrane potential or other factors, which causes excitatory or inhibitory changes in neuronal activity. Neurotransmitters play important roles in the transmission in neuronal circuits related to pain (60). Studies have shown that glutamic acid, *γ*-aminobutyric acid, 5-hydroxytryptamine (serotonin), dopamine, and dynorphin are neurotransmitters closely related to pain emotions (61–64).

At present, the progress of research on pain emotions has expanded from research on low-level single neurotransmitters to research on the structural precision of the interconnections and information transmission of various molecules in neural circuits. It has been found that NP model rats exhibited aggravated depression-like behaviors, accompanied by decreased extracellular signal-regulated kinase and phosphorylation in the hippocampus, decreased nerve regeneration, and short-term synaptic plasticity changes (65). The hippocampus is the key nucleus of pain perception and cognition, and also the key part of emotion regulation. The occurrence of hippocampal neuronal degeneration is considered to be the key pathological link of NP and comorbid mood disorders (66). The amygdala is a brain region involved in pain and emotional-affective states and disorders. One study provided a novel mechanism for elucidating comorbid aversive and depressive symptoms in NP and highlighted that structural and functional neuroplasticity in the amygdala may be important as a promising therapeutic target for comorbid negative emotional-affective disorders in chronic pain (67). The nucleus accumbens, located at the junction of the basal nucleus and the limbic system, is an important part of the reward pathway and plays an important role in the occurrence and development of pain and depression (68, 69), The anterior cingulate cortex has been recognized as a key hub for NP comorbid mood disorder symptoms. The periaqueductal gray matter in the midbrain plays an important role in the descending regulation of nociceptive sensation and regulates emotional behavior (70). The mPFC is critical for both the sensory and emotional/cognitive components of pain (71). Studies have shown that the mPFC is involved in emotion regulation and pain information processing (72). Therefore, studying the functional abnormalities and interactions of these brain regions is of great importance for deepening the understanding of NP and related mood disorders, elucidating their neural bases, and guiding the development of therapeutic strategies.

Although numerous studies have contributed to our understanding of NP-related mood disorders, several challenges and obstacles remain. These include the lack of consensus regarding diagnostic criteria, the absence of reliable assessment tools, limited knowledge on the complex biological mechanisms involved, limited treatment options, and inadequate research sample sizes. To address these issues, future research should focus on establishing consensus diagnostic criteria, developing objective and comprehensive evaluation tools, promoting multidisciplinary collaborations, exploring diverse treatment approaches, conducting large-scale multicenter studies, and establishing mechanisms for data sharing. By addressing these areas, researchers can overcome the current challenges and advance our knowledge and management of NP-related mood disorders.

### 4.3. Advantages and limitations

This study used CiteSpace software for data analysis and visualization, which is an advanced research method. By constructing knowledge graphs and visualizing the evolution of research topics, the knowledge in the field of NP and related mood disorders can be greatly increased, providing decision support for related disciplines. In addition, this study discussed the problems and bottlenecks in this field and put forward some solutions and ideas to provide a reference for follow-up research in this field.

However, there were also some limitations to the present study. First, there were limitations in data collection and collation. Electronic databases were limited to the Web of Science, and other electronic databases such as PubMed, Embase, and the Cochrane Library were not searched and analyzed. In addition, non-English literature was excluded. These may lead to publication bias and language bias. Second, the present research methods and technical analysis could still be improved. This study only used CiteSpace for visual analysis. The results from CiteSpace can provide important guidance, but the limitations of its analysis must be minded. Future research should utilize other methods and techniques, such as with the use of multiple metrological software for comparative analysis, to ensure the comprehensiveness and accuracy of the research results. In future research, it is crucial to clearly state the research conclusions more objectively and cautiously and to explore more research directions and possibilities for innovation to promote the development of research on NP-related mood disorders.

### 4.4. Conclusions

NP is a common and severe chronic pain syndrome, which is often accompanied by mood disorders and increases burdens on families and society. In the present study, bibliometric methods were used to visually analyze the research and academic literature on NP-related mood disorders. The significant increase seen in the number of publications each year showed the growing global importance of this field. The USA was the most influential country with the highest number of publications and highest centrality. Univ Toronto was the most frequently seen and central research institution. The author C GHELARDINI had the highest ranking in terms of publications, and RH DWORKIN was ranked first in terms of cited authors. *PAIN* was a representative journal in this field. Over the past 20 years, the research hotspots in this field mainly included how to improve the efficacy of drugs (such as gabapentin and pregabalin) and how to improve the “quality of life” of patients through clinical trials (such as double-blind and randomized controlled trial). The frontier trends mainly focused on the mechanisms linking “microglia activation”, “oxidative stress”, “neuroinflammation”, and NP-related mood disorders. In the future, research should further strengthen collaborations among countries, institutions, and authors, and conduct in-depth investigations into the mechanisms and influencing factors related to neuropathic pain. Additionally, there is a need to encourage more double-blind clinical trials focusing on drug efficacy and treatment strategies to validate and expand the existing research findings. Furthermore, exploring brain regions is also a crucial direction, as it can help unravel the complex relationship between NP and mood disorders. In summary, this study can help researchers to quickly understand the knowledge structures and current hotspots in this field, provide new directions and ideas for research topics, and contribute to greater research progress.
