# Supplementary material for: Research hotspots and trends on neuropathic pain-related mood disorders: a bibliometric analysis from 2003 to 2023
Source: Front Pain Res (Lausanne). 2023 Dec 21;4:1233444. doi: 10.3389/fpain.2023.1233444 (PMC10764508; doi:10.3389/fpain.2023.1233444)
Supplement: Supplementary file 1 [file Datasheet1.docx]

Supplementary Material

Research Hotspots and Trends on Neuropathic Pain-Related Mood Disorders: A Bibliometric Analysis from 2003 to 2023

**Xiaohua Wang^1†^, Yueyang Zhuang^1†^,** **Zhigang Lin^2,3*^,** **Shuijin Chen^2,3*^, Lechun Chen^2,3^, Hongye Huang^1^, Hui Lin^1^, Shiye Wu^1^**

^1^ College of Rehabilitation Medicine, Fujian University of Traditional Chinese Medicine, Fuzhou, Fujian, China.

^2^ Rehabilitation Hospital affiliated to Fujian University of Traditional Chinese Medicine, Fuzhou, Fujian,China.

^3^ Fujian Key Laboratory of Rehabilitation Technology, Fuzhou, Fujian,China.

^†^These authors contributed equally to this work and share first authorship

*** Correspondence:**

Zhigang Lin

[linzhigang@fjtcm.edu.cn](mailto:linzhigang@fjtcm.edu.cn)

Shuijin Chen

[chenshuijin2023@163.com](mailto:chenshuijin2023@163.com)

# Supplementary Figures and Tables

## Supplementary Figures


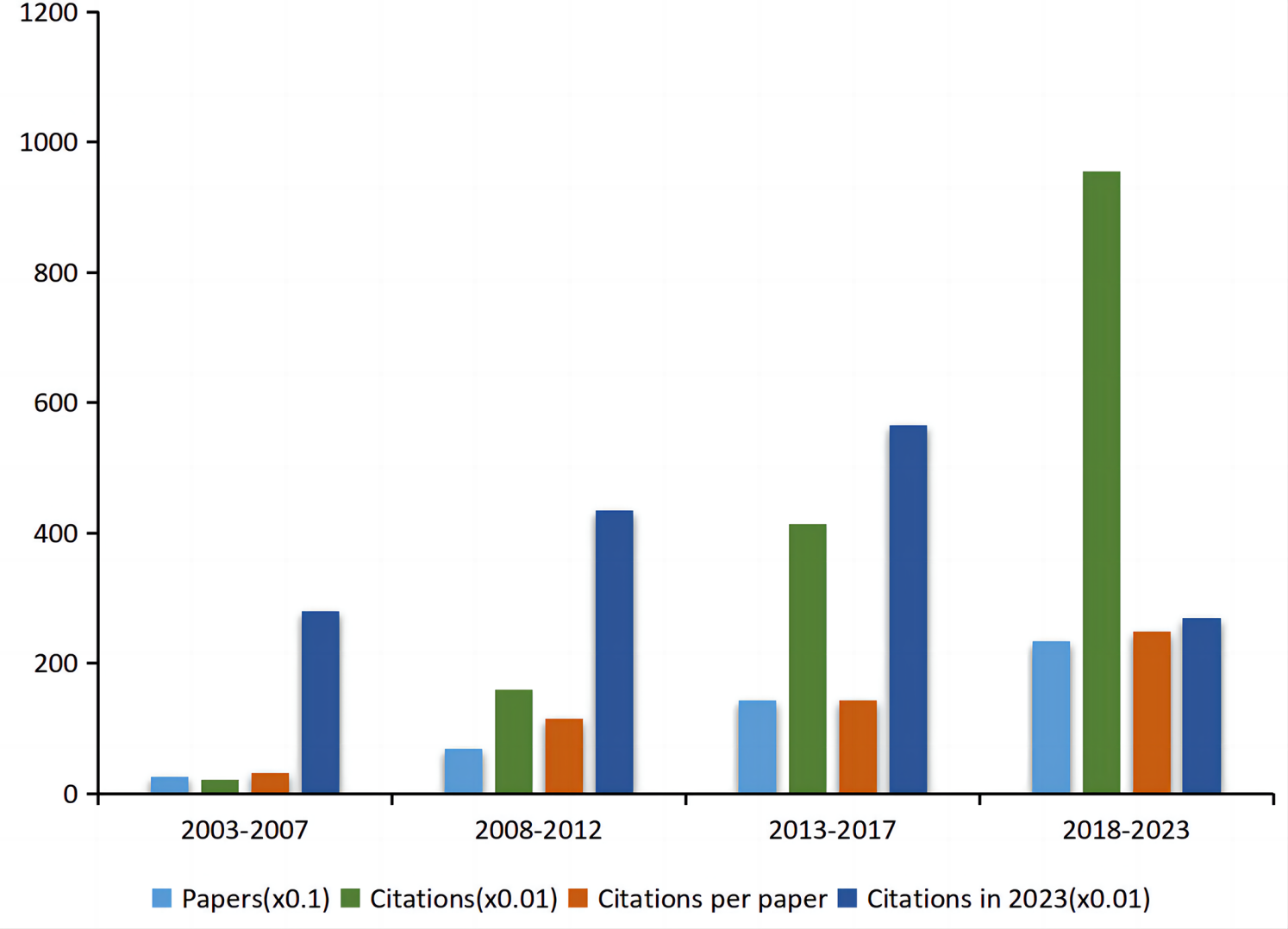


**Supplementary Figure 1.** Number of papers, citations, citations per paper, and citations in 2023 for each 5-year time period.


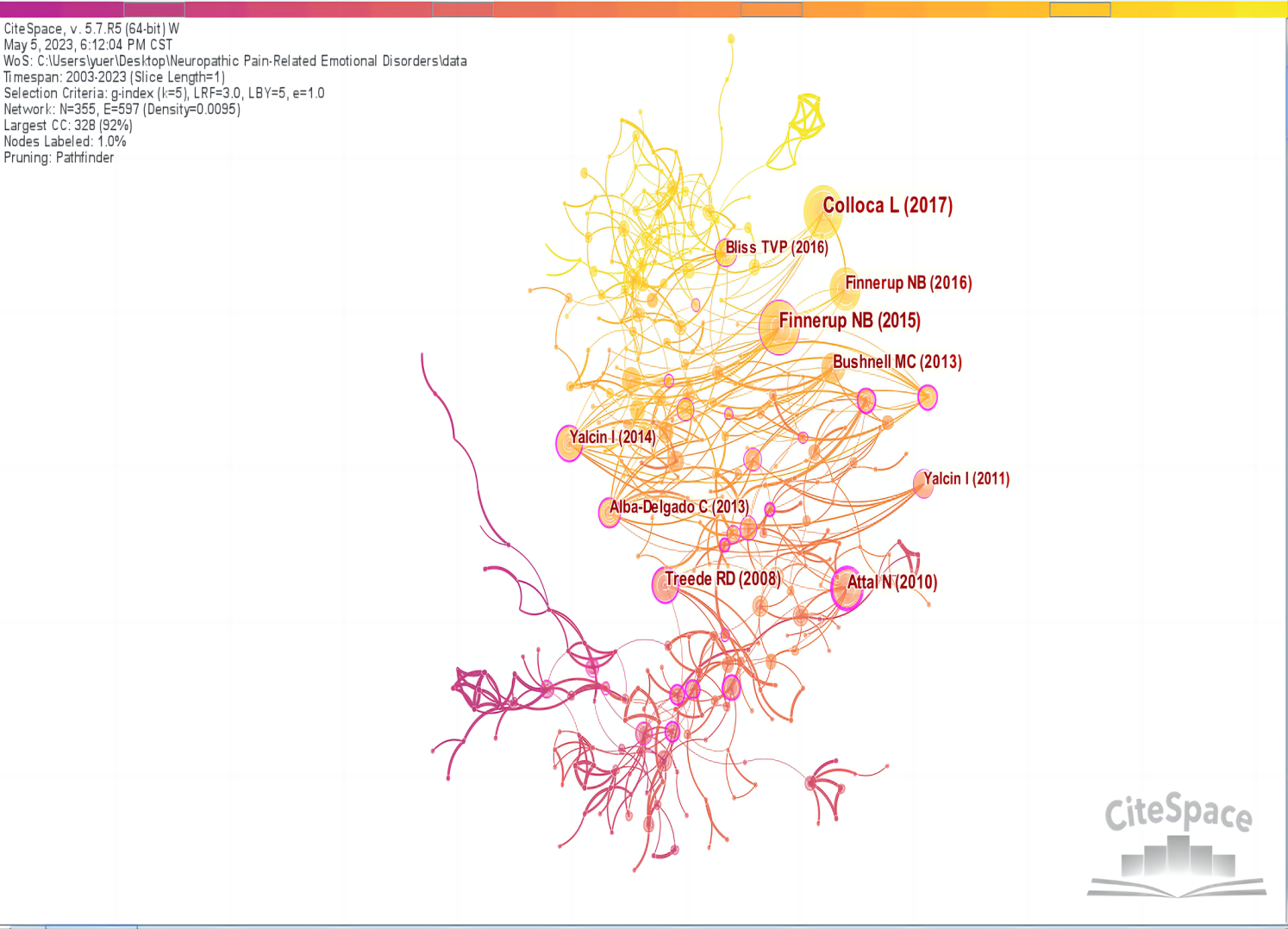


**Supplementary Figure 2.** Map of cited references related to Neuropathic Pain-Related Mood disorders.


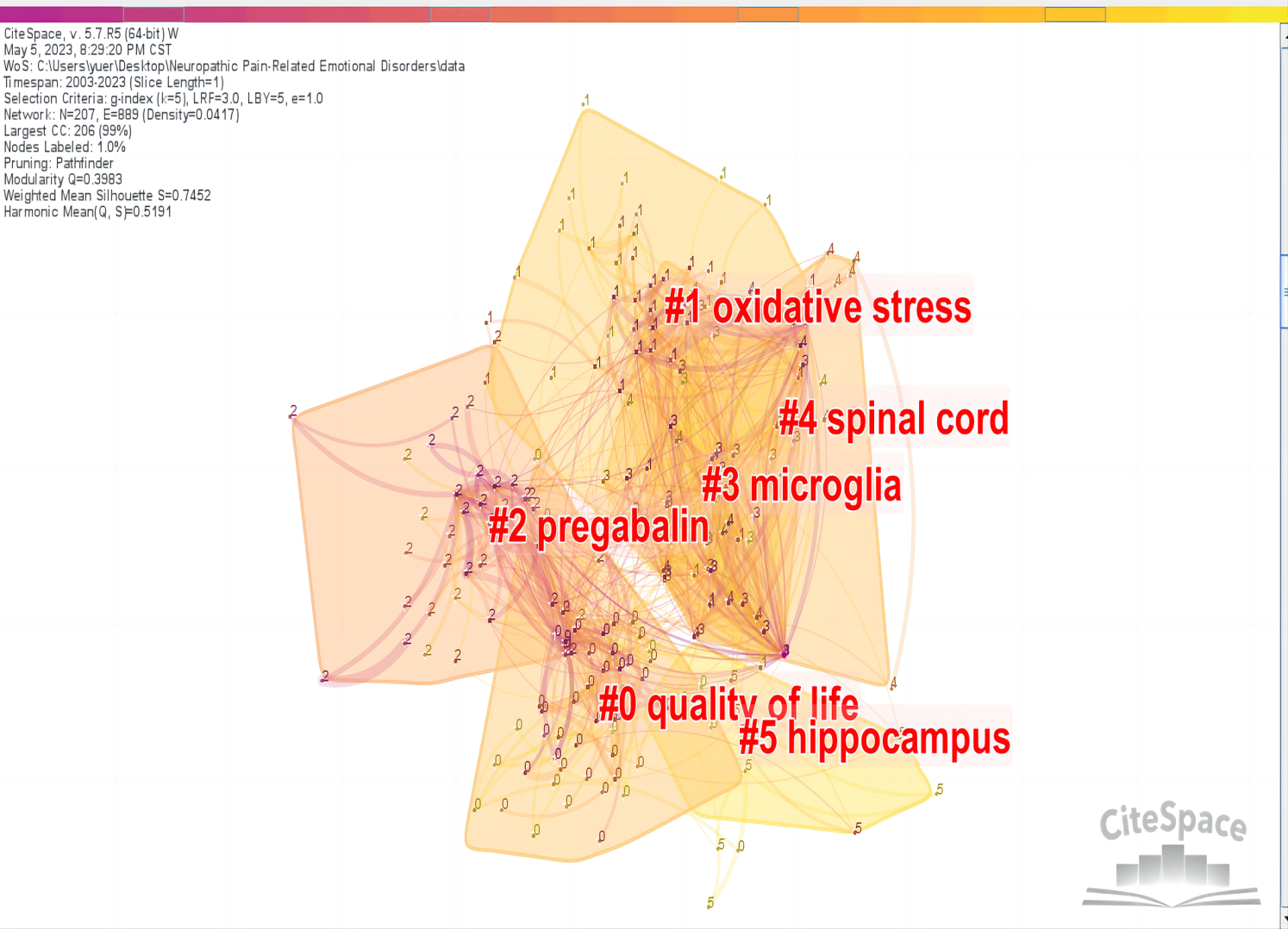


**Supplementary Figure 3.** The cluster map of keywords related to Neuropathic Pain-Related Mood disorders.

## Supplementary Tables

**Supplementary Table 1.** Top 10 frequency of authors.

| Rank | Frequency | Author | Year |
| --- | --- | --- | --- |
| 1 | 20 | CARLA GHELARDINI | 2013 |
| 2 | 16 | MIN ZHUO | 2014 |
| 3 | 16 | LORENZO DI CESARE MANNELLI | 2018 |
| 4 | 15 | OLGA POL | 2018 |
| 5 | 12 | ANDREW S C RICE | 2007 |
| 6 | 12 | ROLFDETLEF TREEDE | 2009 |
| 7 | 11 | DIDIER BOUHASSIRA | 2011 |
| 8 | 10 | JING WANG | 2015 |
| 9 | 9 | GERARD BATALLE | 2021 |
| 10 | 8 | JAVIER REJAS | 2007 |

**Supplementary Table 2.** Top 5 frequency and centrality of cited authors.

| Rank | Frequency | Cited Author | Rank | Centrality | Cited Author |
| --- | --- | --- | --- | --- | --- |
| 1 | 411 | DWORKIN RH | 1 | 0.29 | WOOLF CJ |
| 2 | 403 | CHAPLAN SR | 2 | 0.20 | DWORKIN RH |
| 3 | 338 | BENNETT GJ | 3 | 0.17 | CHAPLAN SR |
| 4 | 323 | FINNERUP NB | 4 | 0.17 | ATTAL N |
| 5 | 320 | BOUHASSIRA D | 5 | 0.13 | BENNETT GJ |

**Supplementary Table 3.** Top 10 frequency and centrality of cited journals.

| **Rank** | **Frequency** | **Cited Journal** | **Centrality** |
| --- | --- | --- | --- |
| 1 | 2912 | PAIN | 0.34 |
| 2 | 1636 | J NEUROSCI | 0.18 |
| 3 | 1427 | J PAIN | 0.04 |
| 4 | 1315 | EUR J PAIN | 0.01 |
| 5 | 1221 | NEUROSCIENCE | 0.07 |
| 6 | 1148 | NEUROSCI LETT | 0.04 |
| 7 | 1130 | BRAIN RES | 0.10 |
| 8 | 1106 | PLOS ONE | 0.02 |
| 9 | 1104 | P NATL ACAD SCI USA | 0.08 |
| 10 | 1060 | EUR J PHARMACOL | 0.07 |

**Supplementary Table 4.** The top 10 cited references.

| **Rank** | **The title of reference** | **Year** | **Cited number** | **Journal** | **Impact factor** | **Quartile** | **Centrality** |
| --- | --- | --- | --- | --- | --- | --- | --- |
| 1 | Neuropathic pain | 2017 | 72 | Nat Rev Dis Primers | 65.038 | Q1 | 0.04 |
| 2 | Pharmacotherapy for neuropathic pain in adults: a systematic review and meta-analysis | 2015 | 65 | Lancet Neurol | 59.935 | Q1 | 0.16 |
| 3 | EFNS guidelines on the pharmacological treatment of neuropathic pain: 2010 revision | 2010 | 41 | Eur J Neurol | 6.288 | Q1 | 0.47 |
| 4 | Neuropathic pain: redefinition and a grading system for clinical and research purposes | 2008 | 39 | Neurology | 11.800 | Q1 | 0.25 |
| 5 | Cognitive and emotional control of pain and its disruption in chronic pain | 2013 | 38 | Nat Rev Neurosci | 38.755 | Q1 | 0.02 |
| 6 | Neuropathic pain: an updated grading system for research and clinical practice | 2016 | 34 | Pain | 7.926 | Q1 | 0.05 |
| 7 | Emotional consequences of neuropathic pain: insight from preclinical studies | 2014 | 32 | Neurosci Biobehav Rev | 9.052 | Q1 | 0.22 |
| 8 | Chronic pain leads to concomitant noradrenergic impairment and mood disorders | 2013 | 29 | Biol Psychiatry | 12.810 | Q1 | 0.26 |
| 9 | Synaptic plasticity in the anterior cingulate cortex in acute and chronic pain | 2016 | 27 | Nat Rev Neurosci | 38.755 | Q1 | 0.16 |
| 10 | A time-dependent history of mood disorders in a murine model of neuropathic pain | 2011 | 27 | Biol Psychiatry | 12.810 | Q1 | 0.12 |

**Supplementary Table 5.** Top 10 frequency and centrality of keywords.

| **Rank** | **Frequency** | **Keyword** | **Rank** | **Centrality** | **Keyword** |
| --- | --- | --- | --- | --- | --- |
| 1 | 2013 | neuropathic pain | 1 | 0.23 | neuropathic pain |
| 2 | 657 | depression | 2 | 0.18 | quality of life |
| 3 | 517 | pain | 3 | 0.17 | double blind |
| 4 | 469 | oxidative stress | 4 | 0.14 | depression |
| 5 | 374 | anxiety | 5 | 0.14 | prevalence |
| 6 | 358 | mechanism | 6 | 0.11 | efficacy |
| 7 | 350 | rat | 7 | 0.10 | oxidative stress |
| 8 | 325 | activation | 8 | 0.09 | pain |
| 9 | 319 | chronic pain | 9 | 0.09 | mechanism |
| 10 | 283 | model | 10 | 0.08 | anxiety |
